# Hemodynamics-guided therapy for hypertensive disorders of pregnancy: a systematic review

**DOI:** 10.1007/s00404-026-08316-3

**Published:** 2026-01-14

**Authors:** Anne-Christin Loheit, Charlotte Lößner, Ekkehard Schleussner, Tanja Groten

**Affiliations:** 1 Member of the Center for Early Pregnancy and Reproductive Health (CEPRE), Obstetric Clinic, University Hospital Jena, Am Uniklinikum 1, 07747 Jena, Germany; 2 Obstetric Clinic, University Hospital Cologne, Cologne, Germany

**Keywords:** Hypertensive disorders of pregnancy, Review, Hemodynamics-guided therapy

## Abstract

**Objective:**

Despite on-going research into the underlying pathology of hypertensive disorders in pregnancy, maternal mortality is hardly decreasing. Current antihypertensive therapy is aimed at controlling symptoms and preventing severe courses of pregnancy. Traditional management focuses on blood pressure (BP) control, but does not consider individual hemodynamic variations. Hemodynamically guided therapy offers a personalized approach that can improve BP control and outcomes by treating the underlying pathophysiology. The aim of this review is to provide an overview of hemodynamically guided antihypertensive therapy in hypertensive disorders of pregnancy and to present the results of recent intervention trials in this area.

**Methods:**

Literature searches were conducted in the electronic databases PubMed, CENTRAL, and Google scholar from inception to May 2024 for studies that used maternal hemodynamic parameters like cardiac output (CO) or TPVR (total peripheral vascular resistance) to guide antihypertensive therapy in pregnant women with hypertensive disorders or at elevated risk for developing preeclampsia. The review included intervention studies.

**Results:**

A total of five studies met the inclusion criteria. All studies showed improved BP control when the antihypertensive medication administered was matched to hemodynamic characteristics of the women being treated. Using a personalized approach, pregnancy complications were significantly reduced in patients with both hypo- or hyperdynamic circulation, even in patients with a history of preeclampsia.

**Conclusion:**

The study shows that hemodynamically triggered antihypertensive therapy can improve outcomes for both mother and child in cases of hypertensive pregnancy disorders. However, further placebo-controlled studies are necessary before a final assessment of this therapy can be made.

**Supplementary Information:**

The online version contains supplementary material available at 10.1007/s00404-026-08316-3.

## Introduction

Despite on-going research into the underlying pathology of hypertensive disorders of pregnancy, maternal mortality is hardly decreasing [1, 2] and still accounts for 6.5% of all pregnancy-related deaths in the US [2]. 5–10% of all pregnant women are affected [3] and the incidence increased over the last 30 years [4]. If hypertension in pregnancy is accompanied by any organ dysfunction, preeclampsia is diagnosed. There are two types of preeclampsia: early-onset (before 34 weeks of gestation) and late-onset (after 34 weeks of gestation). Both types are characterized by different hemodynamic profiles. In early-onset preeclampsia, total peripheral vascular resistance (TPVR) is high and cardiac output (CO) is low (*hypodynamic circulation*), whereas in late-onset preeclampsia, TPVR is low and CO is high (*hyperdynamic circulation*) compared to normotensive pregnant women [5]. Recent studies show that it is not the timing of preeclampsia (early vs. late) that is decisive for these different hemodynamic profiles, but whether the pregnancy is accompanied by fetal growth restriction (FGR) [6]. The causes for the development of preeclampsia are still poorly understood. Like others, Redman and colleagues hypothesize that placental failure in early-onset preeclampsia and premature placental insufficiency due to maternal predisposing factors are associated with late-onset preeclampsia [7]. Consistent with this, other authors assume that pre-conceptual maternal cardiovascular and endothelial dysfunction determines implantation success and the severity of preeclampsia [8]. In addition to short-term adverse maternal and fetal outcomes, the occurrence of preeclampsia has long-term effects on the woman’s cardiovascular health [9–12]. Women after hypertensive disorders of pregnancy are at increased risk of cardiovascular events and coronary artery disease [13–15]. Current antihypertensive therapy is aimed at controlling symptoms and preventing severe courses [16, 17] to prolong pregnancy for the benefit of the fetus. The CHIPS Study [16] showed that tight blood pressure (BP) control (diastolic BP below 85 mmHg) in pregnancy prevents severe hypertension without affecting neonatal outcome in the tight vs. less tight (diastolic BP below 100 mmHg) BP groups. In the CHAP study [17], 2,408 women with chronic hypertension received antihypertensive treatment either immediately (tight) or only if the BP exceeded the cut-off values of systolic 160 mmHg or diastolic 105 mmHg (less tight). The rates of preeclampsia, preterm births, and children born weighing < 2,500g were reduced in the tight group. Both studies support that tight BP control in pregnancy does not affect fetal well-being. However, good BP control is often not achieved and preterm birth is the consequence [17, 18].

## Objectives

Traditional management focuses on BP control, but does not consider individual hemodynamic variations. Hemodynamically guided therapy offers a personalized approach that can improve BP control and outcomes by treating the underlying pathomechanisms. Hemodynamic measurements include various parameters such as TPVR, CO, and BP itself [19–23]. These parameters can be measured using various methods, such as echocardiography (ultrasound of the heart) [21, 24], Impedance cardiography [25, 26] or bioreactance [27, 28]. There are already several non-invasive, user-friendly devices that can easily measure CO and TPVR. These include among others the USCOM and Vicorder device. The USCOM device [29] (Ultrasound Cardiac Output Monitor, Uscom Limite, Australian) is an advanced, non-invasive measuring device for assessing heart function and blood flow. It uses Doppler ultrasound to measure and monitor certain parameters of the cardiovascular system. The Vicorder [30, 31] (Skidmore Medical Ltd, Bristol, United Kingdom) is a medical device used for non-invasive assessment of vascular function and blood pressure measurement. It provides a simple and rapid method for measuring arterial vascular resistance, vascular stiffness, and other hemodynamic parameters.

Based on hemodynamic data, patients can be classified into different subtypes of hypertensive pregnancy disorders, e.g., those with high CO and low SVR or vice versa [5, 19, 22, 23, 32]. The individual hemodynamic parameters are used to select appropriate antihypertensive treatment. This should lead to faster and more effective antihypertensive therapy and improve the outcomes of mother and child.

The aim of this review is to provide an overview of hemodynamically guided antihypertensive therapy in hypertensive disorders of pregnancy and to present the results of recent intervention trials in this area.

## Methods

### Eligibility criteria

Our review included intervention studies that used maternal hemodynamic parameters like CO or TPVR to guide antihypertensive therapy in pregnant women with hypertensive disorders or at elevated risk for developing preeclampsia compared to control groups. We excluded animal studies, case reports, reviews, meta-analyses, conference abstracts, commentaries, and editorials. Studies without a control group or in non-pregnant women were also excluded. There were no restrictions on language or publication date.

### Search strategy

The review protocol adhered to the guidelines in the Preferred Reporting Items for Systematic Review and Meta-analysis Protocols (PRISMA-P) [33]. We performed our advanced research in the electronic databases PubMed, CENTRAL, and Google scholar. Our review included publications from the inception of the databases until 29 May 2024 (Google scholar and PubMed) and 30 May 2024 (Central) and consisted of the following terms and synonyms: “hypertensive disorders of pregnancy”, “gestational hypertension”, “preeclampsia”, “hemodynamic measurements”, “antihypertensive therapy”, “hemodynamically guided therapy”, “noninvasive hemodynamic monitoring”, “pregnancy” (Appendix 1). The protocol was registered with the International Platform of Registered Systematic Reviews and Meta-Analysis Protocols (Registration: INPLASY, INPLASY202560083).

### Study selection and data extraction

All titles and abstracts resulting from our search were screened independently by two reviewers. Based on our selection criteria, eligible studies were identified, and full texts were retrieved. Duplicates were, therefore, excluded. Furthermore, we hand-searched all references of the selected articles and related articles to identify additional relevant publications. Any disagreement was resolved by consensus and, if necessary, a third reviewer was consulted.

### Quality assessment and risk of bias

The quality of data collection and the information on following methodological measures were assessed independently by two reviewers: study design, randomization, blinding of results, reasons for study discontinuation. Disagreements were resolved by consensus and, if needed, a third author was consulted.

The Jadad scale [34] was used to evaluate the methodological quality. According to Jadad scale, studies scoring less than three points were assessed as of low quality.

### Data analysis

Data from the included studies were pooled to provide a narrative synthesis of the findings. Percentages and *p* values are given when presenting statistical results. Due to the limited number of studies, individual studies are discussed in detail in the results section. In our discussion section, other studies in the field of hemodynamic measurements in pregnancy that did not meet the inclusion criteria of our review are briefly mentioned.

## Results

### Study selection and study characteristic

**Fig. 1:**
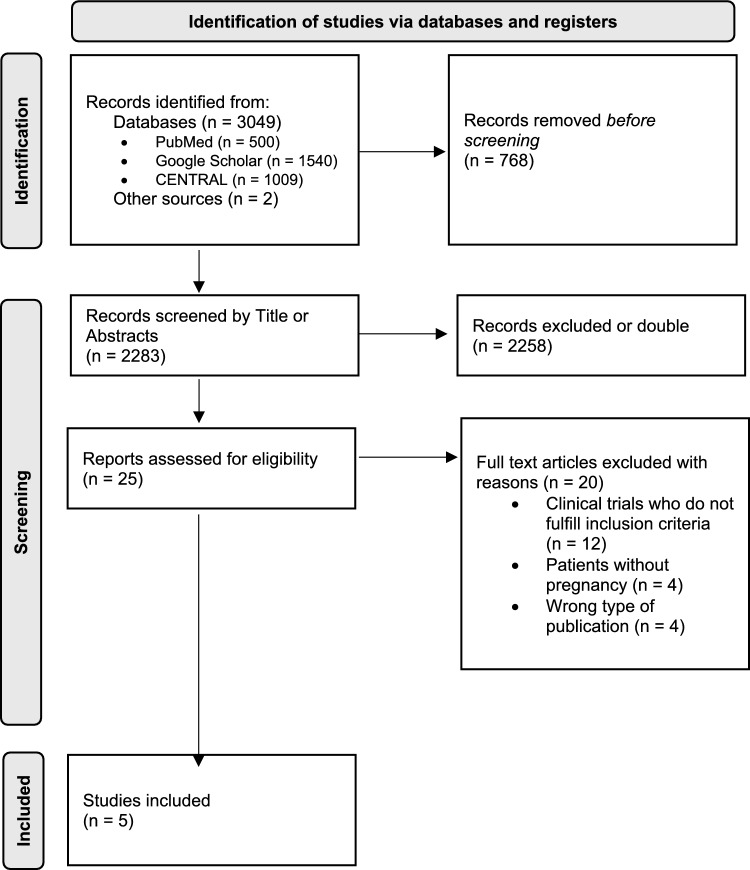
PRISMA flow: the diagram shows the flow of study identification and selection

The PRISMA flowchart (Fig. 1) shows the assessment process in all steps. A total of five studies met the inclusion criteria and were included in our review. All trials were interventional trials. A total of 1,261 pregnant women were included in all trials. Two studies used TPVR [19, 23] as a decision aid for antihypertensive therapy, two used TPVR and CO [21, 22] and one study used CO only [20]. Detailed information on the characteristics of the included studies and the quality assessment is given in Table 1.

**Table 1:** Detailed information on characteristics of included studies and quality assessment

Author and year of publication	Study design	Study population (n)	Inclusion criteria	Exclusion criteria	Gestational age at the time of measurement (weeks of gestation)	Hemodynamic measurement method and parameters recorded	Therapeutic trial/medication	Primary outcome	Results*	Quality according to Jadad Scale**
Vasapollo et al., 2023 [19]	Interventional study	Controls: 160 Cases: 161	Pregnant women with chronic HT and hypodynamic circulation at time of measurement	Tobacco consumption, medical issues other than chronic HT	24	TTE (TPVR)	Controls: SC (methyldopa + nifedipine or amlodipine) Cases: SC + NO-patches of glycerol trinitrate 19 mg/24 h (12 h per day wearing)	Reduction in the occurrence of following outcome parameters (either alone or in combination): - superimposed PE - preterm delivery (before 34 weeks of gestation) due to uncontrolled BP - abruptio placentae - HELLP syndrome - FGR - perinatal death	Controls: 130 (82%) Cases: 86 (53%) p = 0.001	0
Mulder et al., 2021 [21]	Interventional study	Controls: 157 Cases: 157	Women with a history of PE	Not described	Preconceptional, 12, 16, 20, 30	TTE (CO/ TPVR/ BP)	Controls: SC (no details described) Cases: labetalol or methyldopa or nifedipine (treatment before onset of HT)	Occurrence of recurrent PE (defined as new-onset HT with BP ≥ 140/90 mmHg in two repeated measurements together with de novo proteinuria (≥ 0.3 g/24 h or ≥ 2 + on dip- stick analysis) or at least one other maternal organ dysfunction with onset after 20 weeks of gestation in previously normotensive women or superimposed on chronic HT)	Controls: 34 (22%) Cases: 19 (12%) p = 0.03	1
Pasquo et al., 2024 [22]	Interventional study	Inappropriate group: 36 Appropriate group: 116	Women with singleton pregnancies and untreated gestational HT	Severe PE/ HT, HELLP syndrome, chronic maternal diseases, tobacco or drug consumption, fetal anomalies, intrauterine fetal demise, suspected impending fetal compromise, placental abruption, delivery < 48 h from the admission, etc	22–38	USCOM (CO/ TPVR)	Hypodynamic profile: nifedipine/methyldopa (appropriate therapy) Hyperdynamic profile: labetalol (appropriate therapy)	Occurrence of severe HT (defined as systolic BP of ≥ 160 mmHg and/or diastolic BP of ≥ 110 mmHg)	Inappropriate: 7 (19,4%) Appropriate: 7 (6,0%) p = 0.02	1
Easterling et al., 1999 [20]	Double-blind, placebo-controlled interventional study	Controls: 19 Cases: 21	Controls: Nulliparous with CO > 7,4 l/min and Placebo intake Cases: Nulliparous with CO > 7,4 l/min and Atenolol intake	History of severe medical issues	22–25	UltraCom (CO)	Atenolol or placebo if CO > 7,4 l (treatment before onset of HT)	Occurrence of PE (defined as onset of HT and proteinuria)	Placebo: 3 (16%) Cases: 0 (0%) p = 0.04	5
Vasapollo et al., 2012 [23]	Non-randomized trial	Group A: 100 Group B: 100 Group C: 100 Group D: 100	A-D: early-onset mild hypertension, TPVR > 1,350 dyne	Twin pregnancies, undetermined pregnancies, tobacco consumption, maternal medical issues, chromosomal and/ or suspected fetal abnormalities	20–27	TTE (TPVR)	Group A: SC (Nifedipine) Group B: SC + NO donor Group C: SC + oral fluids Group D: SC + NO donor + oral fluids	Progress of mild HT to at least one of the following: - early-onset PE (onset before 34 weeks of gestation) and/or severe PE (associated with FGR, proteinuria > 1 g/24 h, BP > 160/110 mmHg) - therapy-resistant severe gestational HT (rise of BP to > 170/110 mmHg during maximized therapy) - placental abruption - HELLP syndrome - FGR - severe fetal respiratory distress syndrome - perinatal death	Group A: 72 (72%) Group D: 41 (41%) p < 0.001	0

HT, hypertension; PE, preeclampsia; SC, standard care; CO: cardiac output; TTE, transthoracic echocardiography; TPVR, total peripheral vascular resistance; BP, blood pressure; FGR, fetal growth restriction; USCOM, ultrasonic cardiac output monitor

^*^Data are n (%) with indication of significance for the primary outcome if *p* < 0.05 (control group vs. intervention group);

^**^quality assessment according to Jadad scale: 0–2 points: study of poor quality; ≥ 3points: study of high quality;

### Quality assessment and risk of bias

According to Jadad scale, four trials were classified as poor quality [19, 21–23]. Only the study by Easterling et al. was a double-blind, placebo-controlled intervention study and was, therefore, classified as of high quality (Table 2).

**Table 2:** *Quality assessment according to Jadad scale: 0–2 points: study of poor quality; ≥ 3points: study of high quality; **double--blind

	Quality according to Jadad Scale*					
Author and year of publication	Randomization	Blinding**	Description of withdrawals	Randomization/blinding** appropriate	Randomization/blinding** appropriate	Overall
Vasapollo et al., 2023 [19]	0	0	0	0	0	0
Mulder et al., 2021 [21]	0	0	1	0	0	1
Pasquo et al., 2024 [22]	0	0	1	0	0	1
Easterlin et al., 1999 [20]	1	1	1	1	1	5
Vasapollo et al., 2012 [23]	0	0	0	0	0	0

### Synthesis of results

#### Antihypertensive therapy based on TPVR

In a non-randomized study [19], pregnant women with chronic hypertension (CH) and increased TPVR measured at 24 weeks of gestation received nitric oxide donors (NO donors; transdermal NO donors patches of glyceryl trinitrate) and remained on standard therapy (Table 1). This resulted in a prolongation of pregnancies with FGR (p = 0.0001). The rate of maternal and fetal complications in the NO-donor-treated group were lower compared to the standard care group (53% vs 81%; p < 0.001). In 2012, Vasapollo et al. [23] evaluated TVPR in women with early-onset (20–27 weeks of gestation) mild hypertension (defined in this study as a systolic and diastolic BP < 170/110 mmHg) in pregnancy. In that prospective, non-randomized study, a sequential allocation to four different treatment groups was performed (Table 1). Based on TPVR (TPVR > 1,350 dyne), pregnant patients received either standard antihypertensive therapy with nifedipine (group A), or nifedipine and an NO donor (group B), or nifedipine with oral fluids (group C), or nifedipine in addition to an NO donor and oral fluids (group D). The primary outcome was progression from mild hypertension to one or more of the following symptoms: early-onset preeclampsia or severe preeclampsia, severe therapy-resistant hypertension, placental abruption, HELLP syndrome, FGR, severe fetal respiratory distress syndrome or perinatal death. The percentage of patients with severe complications as well as the occurrence of late-onset/mild preeclampsia in group D was lower than in group A (respectively 35% vs 51%; *p* < 0.05; 5% vs 16%, *p* = 0.02).

#### Antihypertensive therapy based on CO

Easterling et al. conducted a double-blind, placebo-controlled intervention study [20] to identify and target hemodynamic abnormalities to further reduce the incidence of preeclampsia. They identified pregnant women at an increased risk of developing preeclampsia by measuring CO to then target treatment before the onset of hypertension. Women with a CO of more than 7.4 l/min were considered as high risk and received either atenolol or placebo (Table 1). The study was not large enough to determine whether its clinical treatment protocol could improve fetal outcomes, but it did show that the rate of preeclampsia was lower in subjects treated with atenolol than in those treated with placebo (0% vs 16% p = 0.04) (Table 1).

#### Antihypertensive therapy based on TPVR and CO

Two studies [21, 22] used TPVR and CO as the basis for antihypertensive therapy. Mulder et al. examined women with a history of preeclampsia and used an algorithm that included BP in addition to CO and TPVR among other parameters to guide therapy decisions. After identifying pregnant women at risk, they selected an antihypertensive drug package based on hemodynamic measurements (Table 1). Labetalol was initiated when heart rate was extremely high in combination with an intermediate-to-low TPVR or when TPVR was extremely low in combination with an intermediate-to-high heart rate. Nifedipine was initiated when heart rate was extremely low in combination with an intermediate-to-high TPVR or when TPVR was extremely high in combination with an intermediate-to-low heart rate. Methyldopa was indicated when both heart rate and TPVR were equally high, without deviating to the extreme sites [21]. Primary outcome was the prevention of recurrent preeclampsia. Secondary outcomes were adverse maternal and offspring outcomes. Normalization of non-physiological hemodynamics in pregnancy halved the risk of recurrent preeclampsia (12% vs 22%, p = 0.03) without adverse effects on fetal outcome. A second non-randomized trial by Pasquo et al. [22] looked at women with untreated gestational hypertension or mild preeclampsia to compare women who received medication according to their hemodynamic findings with those who did not (Table 1). All women had a hemodynamic measurement before starting medication, and the treating physicians were blinded to hemodynamic results. First-line treatment was a vasodilator (nifedipine or alpha-methyldopa) or a beta-blocker (labetalol), according to preference or local protocols. First-line pharmacological treatment was retrospectively adjudicated as hemodynamically appropriate or not. Appropriate therapy was assigned if pregnant women with low CO [≤ 5 L/min] and/or high TPVR [≥ 1,300 dynes/second/cm^2^]) received vasodilators. These are expected to increase heart rate and contractility. For women with high CO [> 5 L/min] and/or low TPVR [< 1,300 dynes/second/cm^2^]), oral labetalol was appropriate. The primary outcome was to compare the occurrence of severe hypertension between women treated with hemodynamically appropriate and inappropriate therapy. The incidence of severe hypertension before delivery was significantly lower in the appropriate therapy group (6.0% vs 19.4%, *p* = 0.02). Moreover, the number of women who achieved target blood pressure within 48 to 72 h of starting treatment was higher in the appropriate treatment group (70.7% vs 50.0%, *p* = 0.02).

## Discussion

### Principal findings and comparison with the existing literature

This review provides an overview of the current evidence on hemodynamic measurements used to guide antihypertensive therapy in hypertensive disorders of pregnancy. All studies showed improved BP control when the antihypertensive medication administered was matched to hemodynamic characteristics of the women being treated. In addition, the progression from mild hypertension to severe hypertension or preeclampsia was reduced. In the present studies, pregnant women at risk of developing preeclampsia benefited from hemodynamically guided therapy, with a significant reduction in the incidence or recurrence of preeclampsia.

The benefit of hemodynamically guided therapy results in terms of outcome and therapy monitoring has previously been demonstrated in non-pregnant subjects with arterial hypertension [35–38]. For example, Taler et al. [36] examined 104 non-pregnant subjects with hypertension and high TPVR measured by thoracic bioimpedance. They then received either standard therapy (selected by the treating physician) or antihypertensive medication according to their hemodynamic profile. They showed superior BP control using a treatment algorithm and serial hemodynamic measurements to guide therapy compared with clinical judgment only. In another trial by Smith et al. [35], impedance cardiography was used to show that hemodynamically guided antihypertensive therapy resulted in greater and more frequent reductions in BP in 164 previously uncontrolled non-pregnant hypertensive patients. A 2014 review of the literature [39] also described a significant reduction in BP in participants treated with impedance cardiography-guided selection of antihypertensive medication compared with standard treatment.

There have been two non-systematic reviews [40, 41] on hemodynamic measurements in pregnancies complicated by hypertensive disorders. Lees et al. [41] concluded that understanding the underlying nature of hypertension may allow for more rational treatment of these conditions, not only to control BP but also to treat the underlying pathology. Hemodynamically targeted atenolol therapy is associated with a blunted rise in maternal sFLT-1 levels during pregnancy [42], and hemodynamically targeted therapy in women at risk was associated with a slower rise in TNF-alpha receptor 1 compared with low-risk untreated women [42]. Studies have shown that antihypertensive medication has an effect not only on BP, but also on hemodynamic pathology in pregnant women [32, 43, 44]. Furthermore, reports have emphasized the feasibility of hemodynamic measurements for the early identification of pregnant women at increased risk of FGR [45]. In addition, hemodynamic parameters have been shown to predict the success of antihypertensive therapy and to identify women who may require prolonged therapy [46, 47]. McLaughlin et al. [40] summarize that non-invasive hemodynamics to guide therapy may be able to better identify the pathogenesis of maternal hypertension, guide appropriate therapy that is safe for the fetus, and prevent adverse maternal and perinatal outcomes.

### Strengths and limitations

The review looks at hemodynamic-based antihypertensive therapy in hypertensive disorders of pregnancy. Only analyses that specifically compared this type of therapy with standard antihypertensive therapy or placebo were included. A limitation of this work was that the included studies did not examine similar parameters, which is why no meta-analysis could be performed. Only one trial was double-blind and placebo-controlled [20]. It should also be mentioned that it is very difficult to conduct blinded placebo-controlled studies during pregnancy. The options for selecting a tool to determine the risk of bias were very limited. There is currently no standardized tool that can represent the risk of bias of all studies included in the review in a comparable way. We, therefore, decided to use the Jadad scale, as it can be used to assess the quality of both randomized and non-randomized studies.

### Recommendations for future research

The results of the present review are promising for hemodynamically guided antihypertensive therapy. However, the small number of current intervention studies in this field does not allow for precise conclusions to be drawn for clinical practice.

Further placebo-controlled intervention trials are needed. In addition, current measurement methods are very static with single measurements over the time. The use of innovative technologies, especially portable devices, has the potential to transform the field of prenatal care. Non-invasive portable devices for measuring hemodynamic parameters in pregnant women represent a growing area in medical technology that has the potential to significantly improve the monitoring and management of maternal health during pregnancy. These devices offer a safe, efficient, and user-friendly method for monitoring important parameters such as blood pressure, heart rate, oxygen saturation, and even uterine blood flow, without the need for invasive procedures or frequent visits to medical facilities [48, 49].

The use of such technologies can be particularly beneficial for monitoring pregnant women at high risk of preeclampsia, gestational diabetes, hypertension, or other conditions that can endanger the mother and unborn child. Through continuous or regular monitoring, these devices enable early detection of potential problems, allowing timely medical intervention to minimize complications during pregnancy and childbirth. Modern wearable technologies use various methods for data collection, including, for example electrodermal activity sensors [50], acoustic sensors [51], pressure sensors [52], photoplethysmography-based sensors [53], or electrocardiographic [54, 55] measurements. These technologies help make the collection of hemodynamic parameters not only more accurate, but also more practical and patient friendly.

However, caution is advised when evaluating and introducing portable non-invasive devices for monitoring hemodynamic parameters in pregnant women. Strict verification of the accuracy, reliability, and safety of these devices must be ensured to avoid erroneous results and the resulting unnecessary anxiety or incorrect treatment. Despite these challenges, these technologies offer great potential for improving prenatal care and protecting the health of mother and child.

### Conclusion

It can be stated that the few intervention studies on hemodynamically guided therapy mentioned show promising results. The study results show that it is perhaps time to change the way we think about and perform antihypertensive therapy during pregnancy, away from a one-size-fits all approach to an individualized therapy that considers the individual hemodynamic constitution of each woman. However, further placebo-controlled studies are necessary before a final assessment of this therapy can be made.

## Supplementary Information

Below is the link to the electronic supplementary material.

Supplementary file1 (DOCX 18 KB)

Supplementary file2 (DOCX 270 KB)

## Data Availability

No datasets were generated or analysed during the current study.
