# Supplementary material for: Hemodynamics-guided therapy for hypertensive disorders of pregnancy: a systematic review
Source: Arch Gynecol Obstet. 2026 Jan 14;313(1):43. doi: 10.1007/s00404-026-08316-3 (PMC12804288; doi:10.1007/s00404-026-08316-3)
Supplement: Supplementary file 1 — Supplementary file1 (DOCX 18 KB) [file 404_2026_8316_MOESM1_ESM.docx]

**Hemodynamics-Guided therapy for hypertensive disorders of pregnancy- a Systematic Review**

| **PubMed**  time frame included in search: inception of databease-05/2024  search: hypertensive disorders of pregnancy OR gestational hypertension OR preeclampsia AND hemodynamic measurements AND antihypertensive therapies OR hemodynamic guided therapy OR Noninvasive Hemodynamic Monitoring AND pregnancy  total hits: 500→ full-text screening: 11 → included: 3 | **Google Scholar**  time frame included in search: inception of databease-05/2024  search: hypertensive disorders of pregnancy, gestational hypertension, preeclampsia, hemodynamic measurements, antihypertensive therapies, hemodynamic guided therapy, Noninvasive Hemodynamic Monitoring, pregnancy  total hits: 1540→ full-text screening: 12→ included: 2 |
| --- | --- |
| **CENTRAL**  time frame included in search: inception of databease-05/2024  search: hypertensive disorders of pregnancy OR gestational hypertension OR preeclampsia AND hemodynamic measurements AND antihypertensive therapies OR hemodynamic guided therapy OR Noninvasive Hemodynamic Monitoring AND pregnancy  (filter: Cochrane Group Pregnancy and Childbirth)  total hits: 1009→ full-text screening: 2 → included: 0 | **Hand search**  full-text screening: 0 →included: 0 |

**Appendix S1:** Databases and search strategies
